# The Effect of Different Immunization Cycles of a Recombinant Mucin1-Maltose-Binding Protein Vaccine on T Cell Responses to B16-MUC1 Melanoma in Mice

**DOI:** 10.3390/ijms21165810

**Published:** 2020-08-13

**Authors:** Hongyue Zhou, Zenan Zhang, Guomu Liu, Mengyu Jiang, Jingjing Wang, Yu Liu, Guixiang Tai

**Affiliations:** Department of Immunology, College of Basic Medical Science, Jilin University, Xinjiang Street 125, Changchun 130021, China; zhouhy18@mails.jlu.edu.cn (H.Z.); zhangzn17@mails.jlu.edu.cn (Z.Z.); 5465511@sohu.com (G.L.); jiangmy18@mails.jlu.edu.cn (M.J.); wangjj17@mails.jlu.edu.cn (J.W.); liuyouyou0809@163.com (Y.L.)

**Keywords:** MUC1-MBP, CpG 2006, antitumor vaccine, immunization cycle

## Abstract

We explored the effect of a recombinant mucin1-maltose-binding protein vaccine, including immunization cycles of recombinant mucin1-maltose-binding protein (MUC1-MBP) and CpG 2006 on T cell responses to human *MUC1*-overexpressing mouse melanoma B16 cells (B16-MUC1) melanoma in mice. We found that the vaccine had a significant antitumor effect, with the most obvious tumor-suppressive effect being observed in mice immunized five times. After more than five immunizations, the tumor inhibition rate decreased from 81.67% (five immunizations) to 43.67% (eight immunizations). To study the possible mechanism, Mucin-1(MUC1)-specific antibodies, IFN-γ secretion by lymphocytes, and cytotoxic T lymphocyte (CTL) cytotoxicity were measured by enzyme-linked immunosorbent assay (ELISA) and a real-time cell analyzer (RTCA). T cell subsets and immunosuppressive cells in the mouse spleen and tumor microenvironment were analyzed by FACS. These results showed that five immunizations activated MUC1-specific Th1 and CTL and reduced the ratio of myeloid-derived suppressor cells (MDSCs) and Th17 in mice more significantly than eight immunizations, indicating that excessive frequency of the immune cycle leads to the increased numbers of immunosuppressive cells and decreased numbers of immunostimulatory cells, thereby inhibiting antitumor immune activity. This data provide an experimental foundation for the clinical application of a recombinant MUC1-MBP vaccine.

## 1. Introduction

Cancer vaccines are designed to amplify tumor-specific T cell responses through active immunization targeting tumor antigen; they are vital tools for effective cancer immunotherapy. Mucin-1 (MUC1) is a tumor-associated antigen (TAA) that is expressed at low levels in normal cells and is highly glycosylated. However, MUC1 is abnormally overexpressed and incompletely glycosylated in tumor cells, making it an ideal target for tumor immunotherapy. To date, various MUC1-targeting tumor vaccines including peptide/protein vaccines, glycopeptides vaccines, DNA vaccines, and dendritic cell (DC) vaccines have been developed. More than sixty such vaccines have entered the clinical trial stage, of which four have entered phase III clinical trials. Peptide/protein vaccines have become a hot area of research, as they are relatively simple, safe and easily produced cancer vaccines [1,2]. However, the disadvantage of peptide/protein vaccines is they do not induce a strong T cell response, which can only be produced in the presence of a suitable adjuvant.

Our previous experiments revealed that maltose-binding protein (MBP) induces the activation of natural killer (NK) cells, macrophages, and Th1 cells, indicating that MBP has potent immune-enhancing activities. Furthermore, we found that MBP induces the maturation and activation of DC through upregulation of costimulatory molecules (CD80, CD86, and MHCII) and promotes Th1 activation through a TLR2-mediated MyD88-dependent pathway and a TLR4-mediated TRIF-dependent pathway [3,4,5,6,7]. Similarly, MBP has been used in a series of vaccines, as it enhances immunogenicity [8]. Therefore, to enhance immunogenicity and uptake by antigen-presenting cells (APC), the human *MUC1* gene was fused with MBP to prepare genetically engineered recombinant protein (MUC1-MBP). Cytotoxic T lymphocyte (CTL) activity is the key to clearing tumor cells and is the gold standard for evaluating the efficacy of tumor vaccines. To enhance T cell responses, a series of adjuvants (CpG ODN, Tα1, BCG, or R848) were screened, all of which can induce T cell responses. We found that MUC1-MBP combined with CpG ODN has the strongest antitumor effect [9]. CpG ODN, a TLR9 agonist, has been widely used as an adjuvant in cancer vaccines, because of its ability to enhance the capacity of Th1-type CD4+ T cell responses and antigen-specific CD8+ T CTL responses. Our previous research results showed that the combined application of CpG 1826 and MUC1-MBP not only increases MUC1-specific antibody production, but also promotes maturation and activation of DC, after which it induces naïve CD4+ T cells to adopt Th1 polarization and enhance MUC1-specific CTL cytotoxicity [9]. CpG 1826, which contains two GACGTT motifs, can specifically activate mouse immune cells, whereas CpG ODN 2006, which contains three GTCGTT motifs, is optimal for human cells. Furthermore, CpG 2006 can activate mouse immune cells [10,11,12], which will allow researchers to use mouse models to study the potential clinical application value of CpG 2006 in the future. Furthermore, CpG 2006 combined with the tumor antigen (NY-ESO-1) induces high levels of CD8+ T cell responses, and CpG 2006 combined with tremelimumab elicits a lasting antitumor response in patients with melanoma and advanced solid tumors [13,14,15,16,17]. Therefore, in the present study, to further optimize the recombinant MUC1-MBP vaccine and make it more suitable for human clinical application, CpG 2006 combined with MUC1-MBP, which was named the recombinant mucin1-maltose-binding protein (recombinant MUC1-MBP) vaccine, was studied. We found that CpG 2006 promoted mouse T lymphocyte proliferation capacity to a level comparable to that induced by CpG 1826 when the dose of CpG 2006 was 4-fold that of CpG 1826 (data not shown). Therefore, human CpG 2006 can be used to study the effects of the recombinant MUC1-MBP vaccine in mouse models.

In the present study, to prepare a human cancer vaccine targeting MUC1, CpG 2006 was used as an adjuvant to improve the immunogenicity of MUC1-MBP. We also explored the antitumor mechanism of the recombinant MUC1-MBP vaccine, mainly focusing on vaccine-induced MUC1-specific Th1 activity and CTL cytotoxicity, as well as the proportion of Th17 and myeloid-derived suppressor cells (MDSCs). Our study highlights the fact that screening of the vaccine immunization cycle is essential for optimizing efficacy, laying the experimental foundation for further clinical study of the vaccine.

## 2. Results

### 2.1. The Recombinant MUC1-MBP Vaccine Inhibited B16-MUC1 Melanoma Growth in a Preventive Mouse Model

**Figure 1:**
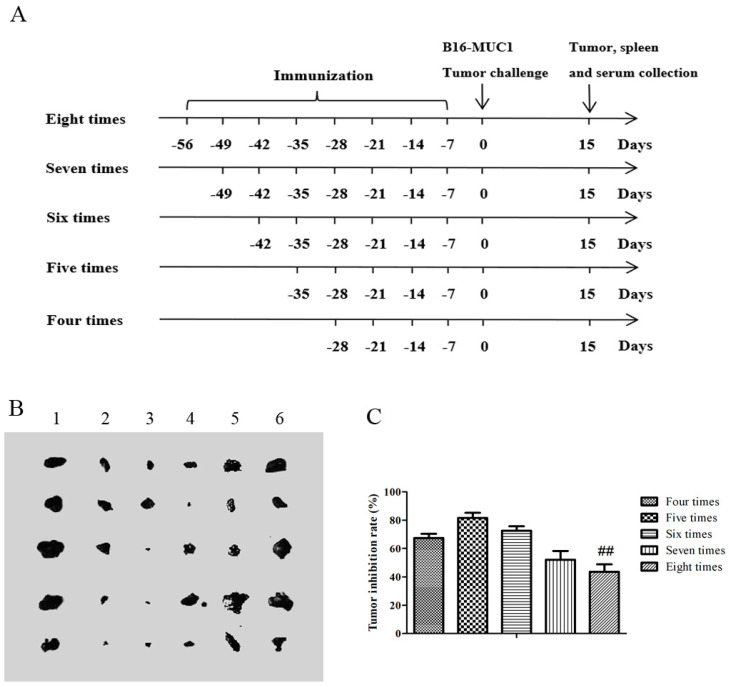
The antitumor effect of the recombinant mucin1-maltose-binding protein (MUC1-MBP) vaccine in the tumor-bearing preventive mouse model. Mice (*n* = 5) received different numbers of subcutaneous immunizations at 7-day intervals, and then were subcutaneous injected (s.c.) with 5 × 10^5^ human *MUC1*-overexpressing mouse melanoma B16 cells (B16-MUC1) melanoma cells one week after the final immunization. The mice were euthanized day 15 after tumor inoculation. (**A**) The experimental workflow of the preventive mouse model. (**B**) The photographs show the tumors dissected from the mice; “1” represents the negative control (NC) group; “ 2, 3, 4, 5, and 6” represent the vaccine groups immunized four, five, six, seven, and eight times, respectively. (**C**) The tumor inhibition rate. Tumor inhibition rate (%) = (1 − experimental group tumor weight/control group) × 100%. ## *p* < 0.01 vs. the group immunized five times.

To explore the optimal immunization cycles of the recombinant MUC1-MBP vaccine including the recombinant MUC1-MBP protein and CpG 2006, mice received different numbers of immunizations, as shown in Figure 1A. One week after the final immunization, the mice were subjected to tumor challenge by subcutaneous injection of human *MUC1*-overexpressing mouse B16 melanoma cells (B16-MUC1). MUC1+ cells clones were 99.7% pure, as verified by flow cytometry (data not shown). In all of the vaccine-immunized mice groups, the tumor sizes were smaller than those in the negative control (NC) group (Figure 1B). In particular, the most obvious tumor suppression was observed in mice immunized with the vaccine five times. After the fifth immunization, subsequent vaccinations weakened the inhibitory effect on the tumor. The tumor inhibition rate decreased from 81.67% (five immunizations) to 43.67% (eight immunizations) (Figure 1C). These results indicated that the recombinant MUC1-MBP vaccine effectively inhibited the growth of B16-MUC1 melanoma, and that antitumor effect was significantly affected by the number of immunization cycles.

### 2.2. Five Immunizations with the Recombinant MUC1-MBP Vaccine Induced Stronger T Cellular Immune Responses than Eight Immunizations in the Preventive Mouse Model

**Figure 2:**
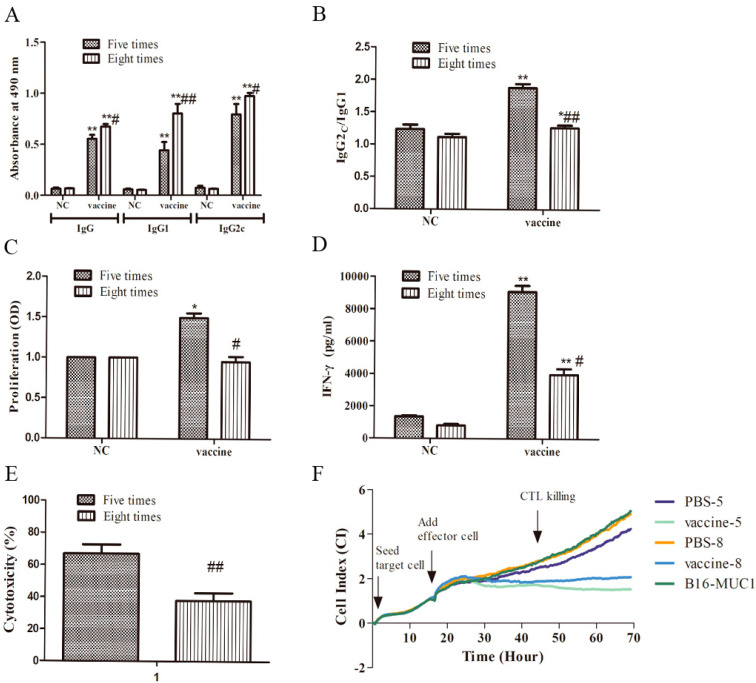
The effect of immunization cycles on the recombinant MUC1-MBP vaccine-induced MUC1-specific humoral and cellular immune responses in the preventive mouse model. (**A**–**F**) Mice (*n* = 5) were immunized with vaccine five or eight times at 7-day intervals, and then subcutaneous injected (s.c.) with 5 × 10^5^ B16-MUC1 melanoma cells one week after the final immunization. The mice were euthanized 15 days after tumor inoculation, and the serum was collected for the MUC1-specific antibody assay. Splenocytes from each group were stimulated in vitro with a specific MUC1 peptide (20 µg/mL) for five days. Then, cell proliferation was measured with the WST-1 kit and cytokine activity was detected by enzyme-linked immunosorbent assay (ELISA). To evaluate cytotoxic T lymphocyte (CTL) killing activity, splenic mononuclear cells from immunized mice stimulated with MUC1 peptide in vitro were used as effector cells, and a B16 mouse melanoma cell line stably expressing human MUC1 was established as target cells. MUC1-specific CTL responses were detected by xCELLigence real-time cell analyzer (RTCA) S16 (**A**) MUC1-specific IgG, IgG1, and IgG2c levels in the sera of the immunized mice were determined using ELISA (**B**) Serum IgG2c/IgG1 ratio. (**C**) Lymphocyte proliferation was measured by the WST-1 assay. (**D**) IFN-γ secretion was measured by ELISA. (E–F) MUC1-specific CTL killing activity was measured by an RTCA. (**E**) CTL killing activity: cytotoxicity (%) = (1 − CI (vaccine group)/CI (NC group)) × 100%. (**F**) The dynamic change in the cell index (CI); PBS-5 and PBS-8 represent effector cells immunized with PBS five and eight times immunizations, respectively; vaccine-5 and vaccine-8 represent effector cells immunized five and eight time immunizations, respectively; B16-MUC1 only represents target cells and no effector cells. * *p* < 0.05, ** *p* < 0.01 vs. the NC group. # *p* < 0.05, ## *p* < 0.01 vs. the group immunized five times.

The results described above showed that an obvious difference in tumor inhibition was observed in mice that received different numbers of immunizations. To study the possible mechanism underlying this difference, the immune response was deeply investigated in mice immunized five and eight times. We analyzed the vaccine-induced T cellular responses, as these responses play a key role in removing tumor cells. In the humoral immune response in C57BL/6 mice, IgG indicates total antibodies, and IgG1 and IgG2c are important subclasses that indicate the Th2-biased and the Th1-biased cellular responses, respectively; therefore, IgG, IgG1, and IgG2c were measured by enzyme-linked immunosorbent assay (ELISA). The results showed that anti-MUC1 antibodies were induced in all the vaccine-immunized mice, but not in PBS-immunized mice (Figure 2A). Furthermore, lower levels of anti-MUC1 IgG (0.5533 versus 0.6732), IgG1 (0.441versus 0.8015), and IgG2c (0.7918 versus 0.9719) antibodies, as well as a higher ratio of IgG2c/IgG1 (1.88 versus 1.26) were induced in the mice immunized five times with the vaccine than in those immunized eight times, suggesting that the immunization with the vaccine five times induced weaker humoral immune responses and was biased toward the Th1 type cellular response in mice (Figure 2B). T cellular responses were also studied, including lymphocyte proliferation, Th1 activation, and CTL killing activity. Splenocytes from the mice immunized five times and eight times were stimulated with MUC1 peptide as described in the Materials and Methods section. The culture supernatants were collected on the fifth day, and MUC1-specific lymphocyte proliferation was measured using a WST-1 assay kit. The result showed that compared with that in the NC group, the stimulation index of MUC1-specific lymphocyte proliferation was significantly increased in mice immunized with the vaccine five times but not those immunized eight times (Figure 2C). IFN-γ levels in MUC1-specific T cells were measured in the culture supernatants by ELISA. As expected, mice that were immunized five times with the vaccine had a higher IFN-γ production (9083.67 pg/mL vs. 3979.67 pg/mL) than those immunized eight times (Figure 2D), suggesting that the vaccine induced a stronger Th1 type cellular immunity in mice immunized five times. Furthermore, splenic mononuclear cells were collected as effector cells and were stimulated with MUC1 peptide in vitro for five days as described in Materials and Methods section. B16-MUC1 cells were used as target cells. The target cells and effector cells were added to the E-plate successively at an effector-to-target (E:T) ratio of 50:1, and CTL killing activity was observed for approximately 54 h using an xCELLigence real-time cell analyzer (RTCA) S16. The curve in Figure 2F shows the dynamic change in the cell index (CI), which represents a relative change in electrical impedance depending on the proliferation or apoptosis rate of the cultured cells. At the same time point, a higher CI represents higher a cell proliferation rate, suggesting stronger cell activity and weaker CTL activity; by contrast, a lower CI represents a higher apoptosis rate, suggesting a lower cell activity and a stronger CTL activity. Specifically, after the addition of the effector cells, over time, the curve obviously changed. The curve of PBS-immunized groups showed a similar upward trend, indicating that the viability of the B16-MUC1 cells was increased and the effectors from the mice immunized with PBS did not significantly kill B16-MUC1 cells. By contrast, the curve of vaccine-immunized groups showed a downward trend, indicating that B16-MUC1 cells were killed and their viability was reduced. According to the formula for CTL cytotoxicity described in the Materials and Methods section, the CTL cytotoxicity of the groups immunized five times and eight times immunization were 67% and 37.6%, respectively, (Figure 2E), suggesting that CTL cytotoxicity was significantly lower in mice immunized with the vaccine eight times than in those immunized five times. Taken together, these results demonstrated that the recombinant MUC1-MBP vaccine induced MUC1-specific CTL killing activity and Th1 activation and that excessive immunization cycles attenuated the recombinant MUC1-MBP vaccine-induced T cellular immune responses, thereby inhibiting antitumor immunity.

### 2.3. Five Immunizations with the Recombinant MUC1-MBP Vaccine More Significantly Upregulated CD4+ T and CD8+ T cells Both in the Spleen and the Tumor Microenvironment (TME) Than Eight Immunizations in the Preventive Mouse Model

**Figure 3:**
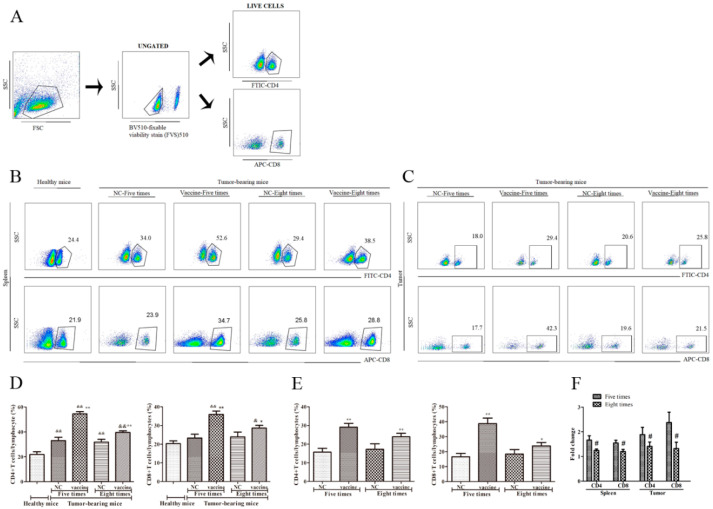
The effect of immunization cycles on recombinant MUC1-MBP vaccine-induced CD4+ and CD8+ T cells in the spleen and the tumor microenvironment. (**A**–**E**) Mice were euthanized 15 days after tumor inoculation and the splenic mononuclear cells from each group were stimulated in vitro with a cocktail (2 µL/mL) for 5 h. Then, the cells were incubated with BV510-conjugated anti-fixable viability stain 510, FITC-conjugated anti-CD4, and APC-conjugated anti-CD8. (**A**) Dot plots showing the gating strategy. (**B**,**C**) The percentages of CD4+ and CD8+ T cells in the spleen and the tumor microenvironment are shown in the dot plots of the flow cytometry data. (**D**,**E**) The percentages of CD4+ and CD8+ T cells in the spleen and the tumor microenvironment are shown in the data table. (**F**) Fold change (CF) represents the ratio of the experimental group to the NC group. Increased fold change = the experimental group/NC group. & *p* < 0.05, && *p* < 0.01 vs. healthy mice. * *p* < 0.05, ** *p* < 0.01 vs. the NC group. # *p* < 0.05 vs. the groups immunized five times.

As previously mentioned, the recombinant MUC1-MBP vaccine significantly enhanced T cell responses in mice. To further study the role of T cell subsets in the antitumor effects of the vaccine, we collected spleen and the tumor samples from all groups 15 days following tumor inoculation. The tumor tissue was digested with collagenase Ⅳ and hyaluronidase, and then cell suspensions of tumor tissue and splenocytes were isolated using EZ-Sep^TM^ Mouse (1×) to obtain Tumor infiltrating lymphocytes (TILs) and splenic mononuclear cells, respectively. Then, CD4+ T and CD8+ T cells subset changes were analyzed by FACS as described in the Materials and Methods section. During cell staining, anti-fixable viability stain 510 Abs were used to remove dead cells, and then live cells were stained with FITC-conjugated anti-CD4 and APC-conjugated anti-CD8 Abs (Figure 3A). The results showed that numbers of the CD4+ T and CD8+ T cells in the spleen were increased in each of the tumor-bearing mice compared with the healthy mice. Moreover, compared with those in the NC group, the numbers of CD4+ T and CD8+ T cells in both the spleen and the TME were significantly increased in the vaccine-immunized mice. More interestingly, more CD4+ T and CD8+ T cells were observed in the mice immunized five times with the vaccine than in those immunized eight times (Figure 3B–E). Specifically, compared with those in the NC group, the numbers of CD4+ T and CD8+ T cells in the spleen of mice immunized five times with the vaccine were increased 1.66-fold and 1.54-fold, respectively, compared to those in control mice, whereas they increased 1.24-fold and 1.2-fold in mice immunized eight times, respectively, compared to those in control mice. In addition, the numbers of CD4+ T and CD8+ T cells in the TME increased 1.86-fold and 2.34-fold, respectively, in mice immunized five times compared to control mice, whereas they increased 1.39-fold and 1.29-fold, respectively, in mice immunized eight times compared to control mice. (Figure 3F). These results suggest that the recombinant MUC1-MBP vaccine enhanced the antitumor effect through increasing the numbers of CD4+ T and CD8+ T cells in the spleen and the TME and that excessive immunization cycles attenuated vaccine-induced CD4+ T and CD8+ T cell responses, thereby inhibiting antitumor immunity.

### 2.4. Five Immunizations with the Recombinant MUC1-MBP Vaccine More Significantly Increased the Th1 and Tc1 Cell Populations and Decreased the Th17 Cell Population in the Spleen Than Eight Immunizations in the Preventive Mouse Model

**Figure 4:**
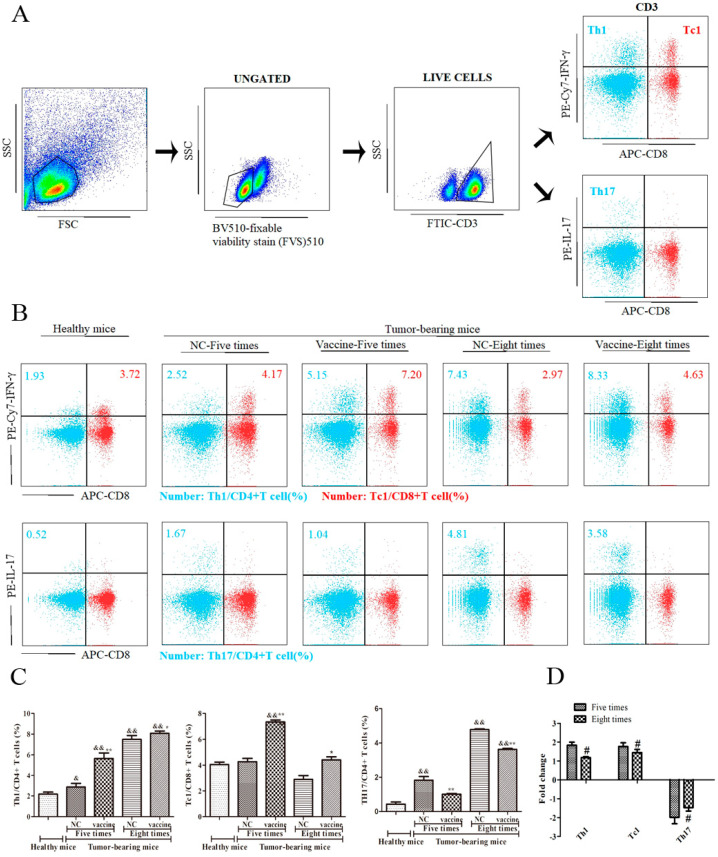
The effect of immunization cycles on recombinant MUC1-MBP vaccine-induced Th1, Tc1, and Th17 cells in the spleen in the preventive mouse model. (**A**–**D**) Mice were euthanized 15 days after tumor inoculation and splenic mononuclear cells from each group were stimulated in vitro with a cocktail (2 µL/mL) for 5 h. Then, the cells were incubated with BV510-conjugated anti-fixable viability stain 510, FITC-conjugated anti-CD3, antigen-presenting cells (APC)-conjugated anti-CD8, PE/CY7-conjugated anti-IFN-γ, and PE-conjugated anti-IL-17 Abs. (**A**) Dot plots showing the gating strategy. (**B**) The percentages of Th1, Tc1, and Th17 cells in the spleen are shown in the dot plots of the flow cytometry data. (**C**) The percentages of Th1, Tc1, and Th17 cells in the spleen are shown in the data table. (**D**) Fold change (CF) represents the ratio of the experimental group to the NC group. Increased fold change = the experimental group/NC group and decreased fold change = − [NC group/the experimental group]. && *p* < 0.01 vs. healthy mice. * *p* < 0.05, ** *p* < 0.01 vs. the NC group. # *p* < 0.05 vs. the group immunized five times.

In antitumor cellular immunity, CD8+ effector T cells kill tumor cells via toxic effector molecules (IFN-γ, perforin, and granzyme), and Th1 helps CD8+ effector T cells differentiate into effector memory cells for long-term immune memory; however, the role of Th17 cells in cancer remains controversial. To further analyze the activity of T cell subsets, splenic mononuclear cells were collected and dead cells were removed as described previously. Then, live cells were stained with FITC-conjugated anti-CD3, APC-conjugated anti-CD8, PE/Cy7-conjugated anti-IFN-γ, and PE-conjugated anti-IL-17 Abs (Figure 4A). Change in CD3+CD8-IFNγ+ T cells, CD3+CD8+IFNγ+ T cells, and CD3+CD8-IL-17+ T cells subsets were detected by FACS. The results showed that the percentage of CD3+CD8-IFNγ+ T cells was increased in each of the tumor-bearing mice groups compared with the healthy mice, whereas the percentage of CD3+CD8+IFNγ+ T cells only increased in mice immunized five times with the vaccine. Moreover, compared with those in the NC group, the percentage of CD3+CD8-IFNγ+ T cells and CD3+CD8+IFNγ+ T cells increased 1.83-fold and 1.72-fold, respectively, in mice immunized five times with the vaccine, whereas they increased 1.18-fold and 1.58-fold, respectively, in mice immunized eight times (Figure 4B–D). By contrast, the percentage of CD3+CD8-IL-17+ T cells significantly increased in the spleens of mice immunized with PBS compared with the healthy mice. Moreover, compared with that in the NC group, the percentage of CD3+CD8-IL-17+ T cells decreased 1.89-fold in mice immunized five times with the vaccine, and decreased 1.54-fold in mice immunized eight times (Figure 4B–D). These results indicated that the recombinant MUC1-MBP vaccine enhanced the antitumor effect by increasing the activity of Th1 and Tc1 cells subsets and by decreasing the activity of Th17 cells in the spleen and that excessive immunization cycles attenuated the activity of the vaccine-induced T cell subsets, thereby inhibiting antitumor immunity.

### 2.5. Five Immunizations with the Recombinant MUC1-MBP Vaccine More Significantly Decreased the MDSC Population and Increased the CD8/MDSC Ratio in Both the Spleen and the Tumor Microenvironment Than Eight Immunizations in the Preventive Mouse Model

**Figure 5:**
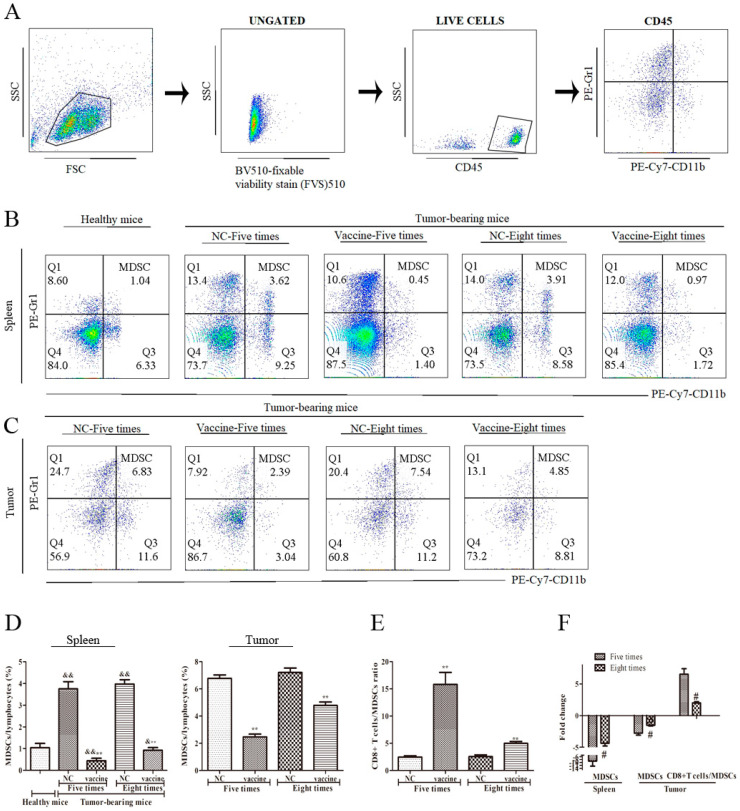
The effect of immunization cycles on the recombinant MUC1-MBP vaccine-induced myeloid-derived suppressor cells (MDSCs) in the spleen and the tumor microenvironment. (**A**–**D**) Mice were euthanized 15 days after tumor inoculation, and splenic mononuclear cells from each group were stimulated in vitro with a cocktail (2 µL/mL) for 5 h. Then, the cells were stained with BV510-conjugated anti-fixable viability stain 510, PE-conjugated anti-Gr1 and PE/CY7-conjugated anti-CD11b Abs. (**A**) Dot plots showing the gating strategy. (**B**,**C**) The percentage of MDSCs in the spleen and the tumor microenvironment is shown in the dot plots of flow cytometry data. (**D**) The percentage of MDSCs in the spleen and the tumor microenvironment is shown in the data table. (**E**) The CD8/MDSC ratio in the tumor microenvironment following immunization with the recombinant MUC1-MBP vaccine. (**F**) Fold change (CF) represents the ratio of the experimental group to the NC group. Increased fold change = the experimental group/NC group and decreased fold change = − [NC group/the experimental group]. && *p* < 0.01 vs. healthy mice * *p* < 0.05, ** *p* < 0.01 vs. the NC group. # *p* < 0.05 vs. the group immunized five times.

Tumor vaccines induce T cell responses to clear the tumor, while tumor cells can induce immunosuppressive cells to evade the attack of immune cells. Myeloid-derived suppressor cells (MDSCs) play an important role in the establishment and maintenance of tumor immune escape [18]. To study the impact of the recombinant MUC1-MBP vaccine on immunosuppressive cell subsets, spleen and the tumor samples were processed, and dead cells were removed as described above. Live cells were stained with PE-conjugated anti-Gr1 and PE/Cy7-conjugated anti-CD11b Abs (Figure 5A), and then CD11b+Gr1+ cells were analyzed by FACS. The results showed that the CD11b+Gr1+ cell percentage in the spleen was significantly increased in tumor-bearing mice immunized with PBS compared with healthy mice. However, compared with that in the NC group, the CD11b+Gr1+ cell percentage in the spleen and the TME was decreased 8.52-fold and 2.75-fold, respectively, in mice immunized five times with the vaccine, whereas it was decreased 4.32-fold and 1.51-fold, respectively, in mice immunized eight times (Figure 5B–D). These results suggested that the recombinant MUC1-MBP vaccine enhanced the antitumor effect by reversing the immunosuppressive state of the TME and that excessive immunization cycles increased the proportion of immunosuppressive cells.

The CD8+ T cells/MDSCs (CD8/MDSC) ratio in the TME has been shown to be related to antitumor immunity [19,20]. The CD8/MDSC ratio reflects the relationship between treatment-induced effectors/suppressors. In our study, compared with that in the NC group, the CD8/MDSC ratio was increased 6.51-fold in mice immunized five times with the vaccine, whereas it was only increased 1.37-fold in mice immunized eight times (Figure 5E,F). This result suggested that excessive immunization cycles attenuated the recombinant MUC1-MBP vaccine-induced CD8/MDSC ratio, reduced vaccine-induced effectors, and increased suppressors, thereby inhibiting antitumor immunity.

### 2.6. Five Immunizations with the Recombinant MUC1-MBP Vaccine Induced T Cell Immune Responses Against B16-MUC1 Melanoma Growth Than Eight Immunizations in the Therapeutic Tumor-Bearing Mouse Model

**Figure 6:**
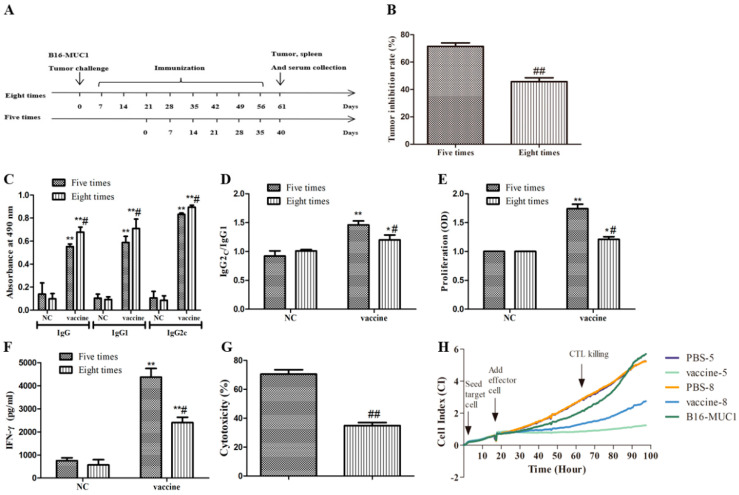
The effect of immunization cycles of the recombinant MUC1-MBP vaccine on the T cell response to B16-MUC1 melanoma in the therapeutic mouse model. (**A**–**H**) Mice (*n* = 5) first were subcutaneously (s.c.) injected with B16-MUC1 melanoma cells on day 0, and then were immunized with the vaccine five or eight times at 7-day intervals. On the fifth day after the final immunization, the mice were euthanized and the tumor, serum, and spleen were collected for the tumor inhibition rate assay, the MUC1-specific antibody, the cell proliferation assay, and the MUC1-specific CTL killing activity assay. (**A**) The experimental workflow of the mouse therapeutic model. (**B**) The tumor inhibition rate. Tumor inhibition rate (%) = (1 − experimental group tumor weight/control group) × 100%. (**C**) MUC1-specific IgG, IgG1, and IgG2c levels in the sera of the immunized mice were determined using ELISA. (**D**) Serum IgG2c/IgG1 ratio. (**E**) Lymphocyte proliferation was measured by the WST-1 assay. (**F**) The IFN-γ secretion was measured by ELISA. (G,H) MUC1-specific CTL-killing activity was measured by an RTCA. (**G**) CTL-killing activity, cytotoxicity (%) = (1 − CI (vaccine group)/CI (NC group)) × 100%. (**H**) The dynamic change in the cell index (CI); * *p* < 0.05, ** *p* < 0.01 vs. the NC group. # *p* < 0.05, ## *p* < 0.01 vs. the group immunized five times.

To better illustrate that the screening of the immunization cycles is important for the recombinant MUC1-MBP vaccine efficacy, a therapeutic mouse model was studied. In the therapeutic model, the mice first were subcutaneously (s.c.) injected with B16-MUC1 melanoma cells on day 0, and then seven days after the tumor challenge, the mice were immunized with the vaccine five or eight times at 7-day intervals, as shown in Figure 6A. On the fifth day after the final immunization, the mice were euthanized; the tumors were removed, and the MUC1-specific antibody levels, lymphocyte proliferation, IFN-γ secretion from lymphocytes, and CTL cytotoxicity were measured. Similar to the findings in the preventive mouse model, these results also showed that five immunizations with the recombinant MUC1-MBP vaccine more significantly inhibited B16-MUC1 melanoma growth than eight immunizations in the therapeutic mouse model, and that this greater inhibition was accompanied by a higher IgG2c/IgG1 ratio, more MUC1-specific lymphocyte proliferation, higher IFN-γ secretion by lymphocytes, and higher CTL cytotoxicity (Figure 6B–H). These results further suggested that excessive immunization cycles attenuated antitumor activity of the recombinant MUC1-MBP vaccine.

## 3. Discussion

In previous studies, we found that CpG 1826 can significantly improve the antitumor growth in mice with a tumor suppression rate of approximately 80% [10]. However, the mouse TLR9 agonist CpG 1826 has a less robust effect in human cells than human CpG 2006, which is a candidate for human clinical trials [12]. Therefore, to make the recombinant MUC1-MBP vaccine more applicable to humans, a recombinant MUC1-MBP vaccine was prepared with recombinant MUC1-MBP protein and CpG 2006. To explore the antitumor mechanism of the vaccine, a prophylactic and therapeutic model of melanoma containing the human *MUC1* gene was used to evaluate the antitumor effect of the vaccine in C57BL/6 mice. First, we screened the immunization cycles of the vaccine. The results showed that after five immunizations, the tumor inhibition rate successively decreased with increasing numbers of vaccinations. In addition, the antitumor effect significantly increased in mice immunized five times compared with those immunized eight times in preventive and therapeutic mouse models. These results suggested that the immunization cycles had a great influence on the antitumor effect of the recombinant MUC1-MBP vaccine. Therefore, to study the possible mechanism, the vaccine-induced immune response was measured in mice immunized five and eight times.

The presence of circulating antibodies against MUC1 correlates with good prognosis in breast cancer patients [21,22]. Our results showed that the recombinant MUC1-MBP vaccine induced high MUC1-specific antibody levels, which were higher in the serum of the mice immunized eight times than in the serum of mice immunized five times in preventive and therapeutic mouse models, suggesting that stronger humoral immune responses were found in mice immunized eight times. This seems to contradict the decline in tumor suppression rate. However, the IgG2c/IgG1 ratio was significantly reduced in mice immunized eight times with the vaccine compared with those immunized five times, suggesting that the Th2 polarizing ability was enhanced and the Th1 polarizing ability was weakened in mice immunized eight times, which may have been related to the reduced tumor suppression rate. Similarly, another article also confirmed that high levels of IgG2/IgG1 but not high levels of IgG1/IgG2 are beneficial for inhibiting tumor growth [23]. Cellular immunity rather than humoral immunity plays a key role in eradicating tumors, and powerful antitumor vaccines need to induce a strong T-cell response and produce lasting immune memory. To further study the role of the recombinant MUC1-MBP vaccine-induced T cell responses on the inhibition of tumor growth, the levels of secreted IFN-γ and the proliferation ability of MUC1-specific cells were measured using ELISA and WST-1 kits, respectively. The results showed that the vaccine increased the proliferation of MUC1-specific cells and the ability of T cells to secrete IFN-γ in the preventive and therapeutic mouse models, suggesting enhanced Th1-type cell responses. Moreover, the vaccine mainly induces Th1 type immune responses, which can help effector CD8^+^ T cells differentiate into memory cells, thereby obtaining lasting immune memory [24]. Therefore, MUC1-specific CTL cytotoxicity was measured using an RTCA. The results showed that the vaccine induced strong antigen-specific CTL killing activity in the preventive and therapeutic mouse models. Consistently, in the preventive mouse model, the results of flow cytometry showed that the vaccine significantly increased the proportion of Th1 and Tc1 cells in the spleen, suggesting enhanced Th1 response and CTL killing activity. Similarly, our previous study found that the recombinant MUC1-MBP vaccine including MUC1-MBP and CpG 1826 also induces significant Th1 responses and CTL killing activity [9]. Furthermore, the results of flow cytometry showed that the vaccine increased the proportion of CD4+ T and CD8+ T cells in the spleen and TME, suggesting the important role of CD4+ T and CD8+ T cells in antitumor immunity. Consistent with our results, Tondini et al. and Tian et al. evaluated CD4+ T and CD8+ T cells in vivo by injecting anti-CD4 and anti-CD8 antibodies in mice, and found that tumor regression was dependent on CD4+ T and CD8+ T cells [25,26,27]. Other experiments have also shown that increases in the numbers of CD4+ T and CD8+ T cells in the TME are closely related to tumor prognosis [28,29,30,31]. In summary, these results indicated that the recombinant MUC1-MBP vaccine inhibited tumor growth by inducing the MUC1-specific T cell response, and that the weakened T cell responses in mice immunized eight times may be related to a decrease in the tumor suppression rate. Consistently, recent reports have shown that antigen-specific T cell responses in cancer patients are the main cause of the antitumor effects of the vaccine [32]. Furthermore, IFN-γ levels, MUC1-specific cell proliferation ability, Th1 and CTL killing activity, as well as the proportion of Th1, Tc1, CD4+ T, and CD8+ T cells were all decreased in mice immunized eight times compared with those immunized five times, suggesting that excessive immunization cycles attenuated the recombinant MUC1-MBP vaccine-induced T cellular immune responses.

Th17 cells are a special subpopulation of CD4+ T cells. IL-17 secreted from Th17 cells is essential for driving inflammation during autoimmune diseases and infections. Th17 has been found in a variety of human cancers; however, the role of Th17 in cancer is highly context-dependent [33]. He et al. showed that administration of IL-17 in tumors inhibits CD8+ T cells infiltration and increases the number of MDSCs [34]. Another article showed that IL-17 can promote tumor growth through the IL-6-STAT3 signaling pathway [35]. However, some reviews have reported that Th17 can inhibit tumor growth. Martin-Orozco et al. showed that Th17 promotes the recruitment of DCs to tumor tissues and significantly activates tumor-specific CD8+ T cells [36]. It has also been shown that Th17 induces the expression of CCL2 and CCL20 in the lung tumor microenvironment and promotes the recruitment of various inflammatory leukocytes (DCs, and CD4α and CD8β T cells) [37]. In our study, in the preventive mouse model, the vaccine significantly reduced the proportion of Th17 cells in the spleen. The ability of the vaccine to reduce Th17 cell activity was lower in mice immunized eight times than in those immunized five times, suggesting that the recombinant MUC1-MBP vaccine may control tumor growth by reducing Th17 and that excessive immunization cycles attenuated the recombinant MUC1-MBP vaccine-induced downregulation of Th17 cells.

When T cells kill tumors, tumor cells escape immune cells by secreting immunosuppressive molecules and recruiting immunosuppressive cells to the tumor site to form an immunosuppressive microenvironment. The major immunosuppressive cells in the TME are MDSCs, which have become important factors leading to tumor progression. Many studies have shown that these cells play key roles in cancer immunosuppression [38,39]. MDSCs can promote the survival and angiogenesis of tumor cells and inhibit the function of T cells by producing the immunosuppressive molecules TGF-β, IL-10, Arg1, and iNOS [40]. MDSCs can induce the chemokines CCL4 and CCL5 to recruit Treg to the tumor sites and cause immunosuppression. To further explore the effect of the recombinant MUC1-MBP vaccine on immunosuppressive cells, MDSCs were detected using flow cytometry in both the spleen and the TME in the preventive mouse model. The results showed that the vaccine significantly reduced the proportion of MDSCs in both the spleen and the TME, and significantly increased the CD8/MDSC ratio, suggesting that the recombinant MUC1-MBP vaccine reversed immunosuppression by reducing MDSCs and thus controlled tumor growth. Similarly, Ma et al. showed that the number of MDSCs in colon cancer patients is negatively correlated with spontaneous MUC1-specific antibody responses in vivo and negatively correlated with T cell proliferation and IFN-γ secretion in vitro [41]. It is worth noting that the ability to reduce the number of MDSCs and increase the CD8/MDSC ratio induced by the vaccine was attenuated in mice immunized eight times, compared with those immunized five times, suggesting that compared with the immunosuppressive MDSCs, CD8+ T cells that inhibited tumor growth were expressed at higher levels, and regulated the immune microenvironment to actively suppress tumor states, suggesting an increase in antitumor activity in mice immunized five times.

Consistent to our results, the phenomenon that the vaccine efficacy was affected by immune cycle were also found in other vaccines. Melanocyte lineage-specific antigen glycoprotein (gp)100 is an ideal target of tumor vaccine. A trial giving up to ten gp100(209-2M) peptide vaccinations, at 3-week intervals, showed that additional vaccinations (more than two immunizations) diminished gp100-specific T cell frequency [42]. In addition, LaCelle et al. found multiple vaccinations of GM-CSF-secreting B16BL6-D5 (D5-G6) melanoma cell line significantly reduced protective antitumor immunity and T cells that mediate regression of established melanoma in adoptive immunotherapy studies. It was associated with an increased frequency and absolute number of CD4+Foxp3+T regulatory (Treg) cells, an immunosuppressive cell which is frequently associated with poor prognosis in cancer [43]. Similarly, Rebecca et al. found that when patients received multiple vaccinations with melanoma peptides in adjuvant IFA, proliferating Treg cells were identified in biopsies [44]. These results suggest that increased numbers of immunosuppressive cells and decreased numbers of immunostimulatory cells limit the efficacy of multiple vaccinations. These findings are consistent with our results, indirectly confirming the rationale of our research. Therefore, for any vaccine to be applied to humans, the issue on the screening of the immune cycle cannot be ignored.

In summary, our study explored the role of the recombinant MUC1-MBP vaccine including the recombinant MUC1-MBP protein and human CpG 2006, elucidating the important role of vaccine-induced T cell subsets in antitumor immunity and screened the immune cycle of the vaccine. The results indicated that the recombinant MUC1-MBP vaccine enhanced MUC1-specific Th1 response and CTL killing activity, and increased the proportion of tumor infiltrating lymphocytes (CD4+ T and CD8+ T cells). In addition, the vaccine also inhibited tumor growth by reducing the number of MDSCs in the TME to reverse the tumor microenvironment. Of note, the ability of the vaccine-induced T cell responses and the ability to inhibit MDSCs were weakened in mice immunized eight times, compared with those immunized five times, suggesting that excessive immune cycles of the recombinant MUC1-MBP vaccine leads to an increase in the number of immunosuppressive cells and a decrease in the number of immunostimulatory cells, thereby inhibiting antitumor immune activity, suggesting that the immune cycle is crucial for vaccine efficacy and that the screening of the best immune cycle of the vaccine is very necessary, laying the theoretical foundation for clinical research on the recombinant MUC1-MBP vaccine.

## 4. Materials and Methods

### 4.1. Cell Lines

B16-MUC1, a human *MUC1*-overexpressed cell line was established in our laboratory from murine B16 melanoma cells transfected with the full-length human *MUC1* gene (22 TR) in the pcDNA3 plasmid as previously described [9]. B16-MUC1 cells were cultured in Iscove’s modified Dulbecco’s medium (IMDM) (Gibco-BRL, Carlsbad, CA, USA) supplemented with 10% fetal bovine serum (FBS, Invitrogen, Carlsbad, CA, USA), antibiotics (100 U/mL penicillin, 100 U/mL streptomycin), and G418 (600 mg/L) (Sigma-Aldrich, St. Louis, MO, USA). B16-MUC1 cells were used to establish a tumor model, in which expression of MUC1 was measured by flow cytometry before each experiment, and MUC1+ cell clone with a purity greater than 98% was prepared for the experiment.

### 4.2. Immunization

Groups of 6- to 8-week-old female C57BL/6 mice (*n* = 5, HFK Bioscience.Co., Beijing, China) were maintained under specific pathogen-free conditions and were immunized with the recombinant MUC1-MBP vaccine including MUC1-MBP (50 μg, prepared by our laboratory) and CpG ODN 2006 (5′- TCGTCGTTTTGTCGTTTTGTCGTT-3′, 75 μg, RiboBio Co. Ltd., Guangzhou, China) for different immunization cycle as shown in Figure 1. In brief, each mouse received a single s.c. injection of 200 μL of PBS containing 50 μg of MUC1-MBP and 75 μg of CpG ODN 2006 into the inguinal lymph node area. Control mice were immunized with 200 μL of PBS. Seven-day intervals were chosen as the immune cycle, which included four, five, six, seven, and eight immunizations. Experimental manipulation of mice was conducted in accordance with the National Institute of Health Guide for the Care and Use of Laboratory Animals and the approval of the Scientific Investigation Board of Science and Technology of Jilin Province (Changchun, China). Studies were performed in accordance with the guidelines established by the Jilin University Institutional Animal Care and Use Committee (approved on 1 January 2016, Protocol No. 2015-34).

### 4.3. Tumor Protection in a Prophylactic Model

The effect of the immunization cycle of the recombinant MUC1-MBP vaccine on T cell responses to B16-MUC1 melanoma in mice was evaluated in a prophylactic and a therapeutic mouse model. The mice were immunized as the previously described. One week after the final immunization, mice were subjected to the tumor challenge by s.c. injection with 5 × 10^5^ B16-MUC1 cells. Fifteen days after tumor challenge, the mice were euthanized, and the tumors were removed. In the therapeutic model, the mice first were subcutaneous (s.c.) injected with 5 × 10^5^ B16-MUC1 melanoma cells on day 0, and then seven days after tumor challenge, the mice were immunized with the vaccine five or eight times at 7-day intervals. On the fifth day after the final immunization, the mice were euthanized, and the tumors were removed.

For MUC1-specific antibodies detection, serum was collected from mice immunized five and eight times; for theMUC1-specific cell proliferation assay and the Th1 activity assay, splenocytes from mice immunized five and eight times were stimulated with MUC1 synthetic peptide (30 amino, Ziyu Biotechnology Co., Ltd., Shanghai, China). For the CTL cytotoxicity and T cell subset assays, splenocytes were isolated using EZ-Sep^TM^ Mouse (1×) (Daktronics Biotech Co., Ltd., Shenzhen, China) from mice immunized five and eight times. Subsequently, the splenic mononuclear cells were stimulated with MUC1 synthetic peptide (30 amino, Ziyu Biotechnology Co., Ltd., Shanghai, China) or cell stimulation cocktail (including protein transport inhibitors, eBioscience, Inc., San Diego, CA, USA). The details were as follows.

### 4.4. ELISA for MUC1-Specific Immunoglobulin Subclasses

MUC1-specific antibodies and antibody subclasses in the mouse serum were measured according to a previously described method [9]. Fifteen days after tumor challenge, serum was isolated from the immunized mice, and MUC1-specific antibodies were measured using enzyme-linked immunosorbent assay (ELISA). Briefly, 96-well plates were coated with 10 µg/well of the MUC1 peptide (30 amino, Ziyu Biotechnology Co., Ltd., Shanghai, China) and incubated at 4 °C overnight. On the second day, the plates were washed four times using PBS-T and were blocked with PBS containing 2% bovine serum albumin for 1.5 h at 37 °C. After washing with PBS-T four times, the serum samples were diluted 1:500 and then incubated at 37 °C for 1.5 h. For IgG, IgG1, and IgG2c antibody titer detection, the plates were washed with PBS-T as previously described and were incubated with horseradish peroxidase-labeled goat anti-mouse IgG, IgG1, and IgG2c (Sigma Chemical Co., St. Louis, MO, USA) for 1 h at 37 °C. The plates were then washed as previously described and were incubated with the substrate o-phenylenediamine dihydrochloride (OPD, Amresco, Solon, OH, USA) for 10 min, and 0.2 mM H_2_SO_4_ was added to terminate the reaction. The absorbance was measured using a microplate reader at a wavelength of 490 nm (BioTek Instruments, Inc., Winooski, VT, USA). The results are expressed as the average value ± standard deviation (SD).

### 4.5. MUC1 Specific Cell Proliferation and Th1 Activity Assay

Fifteen days after tumor challenge, the mice were euthanized; splenocytes were cultured in IMDM containing 100 U/mL interleukin-2 with or without 20 µg/mL MUC1 peptide (30 amino, Ziyu Biotechnology Co., Ltd., Shanghai, China) at a density of 1 × 10^6^ cells/well at 37°C in 5% CO_2_ for five days in 96-well flat bottom plates. The culture supernatants were collected for cytokines assay. Next, for the MUC1-specific cell proliferation assay, WST-1 reagent (Dojindo Molecular Technologies, Tokyo, Japan) was added to each well at a final concentration of 10% *(v/v)*, and the plates were cultured in dark at 37 °C for 1 h. Next, the absorbance was measured using a microplate reader at a wavelength of 450 nm (BioTek Instruments, Inc. Winooski, VT, USA). The results are expressed as relative cell viability. The relative cell viability was calculated as A450 (MUC1-stimulated group)/A450 (control group). For the MUC1-specific Th1 activity assay, IFN-γ production in the culture supernatants was assessed using an ELISA kit (eBioscience, Inc., San Diego, CA, USA) according to the manufacturer’s instructions. MUC1-specific IFN-γ production is expressed as the level of cytokine secreted by splenocytes stimulated by MUC1 peptide minus the number of cytokines secreted by nonspecific stimulated cells.

### 4.6. MUC1-Specific CTL Cytotoxicity Assay

Cytotoxicity was measured using an electrical impedance-based approach, namely, the Real-Time Cell Analyser (RTCA, Roche Applied Science, GmbH, Penzberg, Germany). The system detects variations in electrical impedance across incorporated sensor electrode arrays placed on the bottom of 16-well chamber slide plates (E-plate 16) on which cells are seeded. The main read-out of the RTCA is a dimensionless parameter called cell index (CI), which represents a relative change in electrical impedance, depending on the proliferation or apoptosis rate of the cultured cells. For the MUC1-specific CTL cytotoxicity assay, splenocytes were isolated using EZ-Sep^TM^ Mouse (1×) (Daktronics Biotech Co., Ltd., Shenzhen, China) from immunized mice, and then the splenic mononuclear cells were stimulated with MUC1 peptide (30 amino, Ziyu Biotechnology Co., Ltd., Shanghai, China) for five days. The MUC1-recalled splenic mononuclear cells were used as effectors. The B16-MUC1 cells were used as target cells. First, 50 µL IMDM (Gibco-BRL, Carlsbad, CA, USA) per well was added to E-plates for baseline measurement. Then, 5× 10^3^ B16-MUC1 cells were seeded in E-plates at a volume of 100 µL per well using as target cells, and electrical impedance was measured throughout the cultivation period at 5-min intervals throughout the culture period until the target cells were in logarithmic growth (total time: 16 h). Next, splenic mononuclear cells from vaccine-immunized mice were stimulated in vitro according to a previously described method and were plated at a density of 2.5 × 10^5^ cells/well in E-plates in a volume of 100 µL per well at an effector-to-target cell (E/T) ratio of 50:1. Splenic mononuclear cells from PBS-immunized mice served as negative control (NC). The electrical impedance was measured throughout the cultivation period in 10-min intervals (total time: 54 h or 72 h). The percentage of cytotoxicity was calculated as follows: cytotoxicity (%) = (1 − CI (vaccine group)/CI (NC group)) × 100%.

### 4.7. Isolation of Tumor Infiltrating Lymphocytes (TILs)

To examine tumor infiltrating cells, tumor tissues were cut into small pieces and digested in IMEM (Gibco-BRL, Carlsbad, CA, USA) containing collagenase IV (1 mg/mL) (Suo Laibao Technology Co., Ltd. Beijing, China) and hyaluronidase (0.1 mg/mL) (Suo Laibao Technology Co., Ltd. Beijing, China) for 1 h at 37 °C. Then, cell suspensions of tumor tissues were isolated using EZ-Sep^TM^ Mouse (1×) (Daktronics Biotech Co., Ltd., Shenzhen, China).

### 4.8. Flow Cytometry Analysis

To compare the effects of different number of immunizations with the recombinant MUC1-MBP vaccine on T cell subsets, the proportions of CD4+ T, CD8+ T cells, Th1 (CD3+CD8-IFN-γ+) cells, Tc1 (CD3+CD8+IFN-γ+) cells, Th17 (CD3+CD8-IL17+) cells and MDSCs (CD11b+Gr1+) were measured by flow-cytometry.

For the T cell subset assay, splenocytes were isolated using EZ-Sep^TM^ Mouse (1×) (Daktronics Biotech Co., Ltd., Shenzhen, China). Then, splenic mononuclear cells were stimulated with cell stimulation cocktail (plus protein transport inhibitors, 2 μL/mL, eBioscience, Inc., San Diego, CA, USA, 00-4975-93) for 5 h before staining. Cells (1 × 10^6^) were used as experimental samples. Th1 (CD3+CD8-IFN-γ+) cells, Tc1 (CD3+CD8+IFN-γ+) cells, and Th17 (CD3+CD8-IL17+) cells were stained extracellularly with anti-fixable viability stain 510 (fvs510, BD, San Jose, CA, USA,564406) and anti-CD4 (FITC, BD, San Jose, CA, USA, 553046) and anti-CD8 (APC, eBioscience, Inc., San Diego, CA, USA, 17-0081-81) Abs for 30 min at 4 °C in the dark. Subsequently, the cells were washed with PBS twice, fixed and permeabilized with Perm/Fix solution (eBioscience, Inc., San Diego, CA, USA, 00-5523) according to the manufacturer’s instructions. Then, the cells were intracellularly stained with anti-IL17 (PE, BD, San Jose, CA, USA, 559502) and anti-IFN-γ (PE/Cy7, BD, San Jose, CA, USA, 505826) Abs for 30 min at room temperature in the dark. For CD4+ T, CD8+ T cells, splenic mononuclear cells, and TILs were obtained as previously described and were stained extracellularly with anti-CD4 (FITC, BD, San Jose, CA, USA, 553046) and anti-CD8 (APC, eBioscience, Inc., San Diego, CA, USA,17-0081-81) Abs. For MDSCs, splenic mononuclear cells and TILs obtained as previously described were stained with anti-CD11b (PE/Cy7, BD, San Jose, CA, USA, 561098) and anti-Gr-1 (PE, BD, San Jose, CA, USA, 561084) Abs for 30 min at 4 °C in the dark. Flow cytometry was performed using a BD FACSAria II system (BD, San Jose, CA, USA) and the data were analyzed using FlowJo V10.0 software (TreeStar, Ashland, OR, USA).

### 4.9. Statistical Analysis

One-way analysis of variance (ANOVA) was used to analyze the experimental data. Two-sided Student’s t-test was used to compare the mean values of individual treatments when the primary outcome was statistically significant. *p* < 0.05 was considered statistically significant. All statistical analyses were performed using SPSS 18.0 software (IBM, Amonk, NY, USA).

## Abbreviations

MUC1-MBP	Recombinant mucin1-maltose-binding protein
TAA	Tumor-associated antigen
DC	Dendritic cell
MBP	Maltose-binding protein
APC	Antigen-presenting cells
B16-MUC1	Human *MUC1*-overexpressing mouse melanoma B16 cells
MDSCs	Myeloid derived suppressor cells
CTL	Cytotoxic T lymphocyte
RTCA	Real-time cell analyser
TME	Tumor microenvironment
TILs	Tumor infiltrating lymphocytes
NC	Negative control
CI	Cell index
ELISA	Enzyme-linked immunosorbent assay
CD8/MDSC	CD8+ T cells/MDSCs
NK	Natural killer
MUC1	Mucin-1
